# Discovery of a New Class of Sortase A Transpeptidase Inhibitors to Tackle Gram-Positive Pathogens: 2-(2-Phenylhydrazinylidene)alkanoic Acids and Related Derivatives

**DOI:** 10.3390/molecules21020241

**Published:** 2016-02-19

**Authors:** Benedetta Maggio, Demetrio Raffa, Maria Valeria Raimondi, Stella Cascioferro, Fabiana Plescia, Domenico Schillaci, Maria Grazia Cusimano, Ainars Leonchiks, Dmitrijs Zhulenkovs, Livia Basile, Giuseppe Daidone

**Affiliations:** 1 Dipartimento di Scienze e Tecnologie Biologiche, Chimiche e Farmaceutiche, Sezione di Chimica e Tecnologie Farmaceutiche, Università degli Studi di Palermo, Via Archirafi 32, I-90123 Palermo, Italy; mariavaleria.raimondi@unipa.it (M.V.R.); stellamaria.cascioferro@unipa.it (S.C.); fabiana.plescia@unipa.it (F.P.); domenico.schillaci@unipa.it (D.S.); mariagrazia.cusimano@unipa.it (M.G.C.); giuseppe.daidone@unipa.it (G.D.); 2 IEMEST, Istituto Euromediterraneo di Scienza e Tecnologia, Via Emerico Amari 123, I-90139 Palermo, Italy; 3 Latvian Biomedical Research and Study Centre, Ratsupites 1 k1, 1067 Riga, Latvia; ainleo@biomed.lu.lv (A.L.); dmitry@lu.lv (D.Z.); 4 Dipartimento di Scienze del Farmaco, Università degli Studi di Catania, Viale A. Doria 6 Ed. 2, Città Universitaria, I-95125 Catania, Italy; livia.basile@etnalead.com

**Keywords:** sortase A, biofilms, 2-(2-phenylhydrazinylidene)alkanoic acid derivatives, FRET

## Abstract

A FRET-based random screening assay was used to generate hit compounds as sortase A inhibitors that allowed us to identify ethyl 3-oxo-2-(2-phenylhydrazinylidene)butanoate as an example of a new class of sortase A inhibitors. Other analogues were generated by changing the ethoxycarbonyl function for a carboxy, cyano or amide group, or introducing substituents in the phenyl ring of the ester and acid derivatives. The most active derivative found was 3-oxo-2-(2-(3,4dichlorophenyl)hydrazinylidene)butanoic acid (**2b**), showing an IC_50_ value of 50 µM. For a preliminary assessment of their antivirulence properties the new derivatives were tested for their antibiofilm activity. The most active compound resulted **2a**, which showed inhibition of about 60% against *S. aureus* ATCC 29213, *S. aureus* ATCC 25923, *S. aureus* ATCC 6538 and *S. epidermidis* RP62A at a screening concentration of 100 µM.

## 1. Introduction

Antibiotic resistance is a very important challenge and in 2015 the World Health Organization (WHO) considered it as one of the most important global health problems [1]. The overuse of antibiotics, both in human and animal populations, plays an important part in the appearance of drug-resistant strains.

The drug resistance of Gram-positive pathogens is currently of great significance and in this context *Staphylococcus aureus* that is responsible of both acute and chronic infectious diseases has an extraordinary ability to develop antibiotic-resistance [2]. Its great versatility as a pathogen is due to a huge number of virulence factors [3]. Among the most important virulence factors that it displays during the pathogenesis, the cell-wall associated proteins called microbial surface components recognizing adhesive matrix molecules (MSCRAMMs) can promote the adherence to host tissue by interacting with fibronectin. Other aspects of pathogenesis such as invasion, escape from host defences and the formation of biofilms, that cause chronic infectious diseases or biomaterial associated infections, are also due to the MSCRAMMs [4,5].

Sortase A (SrtA) is the enzyme that incorporates the MSCRAMMs to the peptidoglycan through the following mechanism: the enzyme first cleaves the bond in the sorting signal between the threonine (T) and the glycine (G) residues of a LPxTG motif of cellular proteins; then it causes the formation of a thioester acyl-enzyme intermediate; the last step is a transpeptidation of an amide bond of the carboxyl terminal of threonine and the amine terminus of a pentaglycine cross bridge in peptidoglycan precursors [6].

*S. aureus* strains lacking the SrtA gene do not display surface proteins at the cell wall. Therefore, *SrtA* mutant strains are less virulent than wild strains and they are defective during their pathogenic action [7]. At least twenty different *S. aureus* surface proteins that carry a C-terminal LPxTG motif have been described. These virulence factors include protein A (Spa), two fibronectin binding proteins (FnbpA and FnbpB) and two clumping factors (ClfA and ClfB). Some of these proteins play key roles in biofilm formation [7,8].

An anti-virulence strategy based on agents that target virulence determinants could be effective in preventing the biofilm formation of Gram positive bacteria that are naturally resistant to current antibiotics. Considering that the first crucial step in staphylococcal pathogenesis and biofilm formation is bacterial adhesion, promoted by the surface exposed proteins at the cell wall, we presume that the new inhibitory agents targeting the sortase enzyme that links surface proteins to the cell wall are potentially more useful rather than any single MSCRAMM involved in the pathogenesis [9]. Consequently, sortase A is a good target to develop novel anti-virulence agents and new classes of SrtA inhibitors could tackle the first stage of infectious disease process and biofilm formation [10].

A number of promising small synthetic organic compounds that work as effective SrtA inhibitors and could be developed as anti-virulence drugs, were recently reviewed [11]. Most of classes of described inhibitors (diarylacrylonitriles [12], rhodanines [13], pyridazinones [13], pyrazolethiones [13], 3,6-disubstituted triazolothiadiazol [14], aryl(β-amino)ethyl ketones [15] and benzo-[*d*]isothiazol-3(2*H*)-one adamantanes [16], were identified by high-throughput screening (HTS) or virtual screening. SrtA is an ideal target not associated with bacterial growth and cell death, but rather related to virulence [17]. Moreover, as opposed to conventional antibiotics, in response to which the evolution of resistance by the pathogens is advantageous and nearly unavoidable, in the case of antivirulence agents, the bacterial resistance is potentially costly and therefore less probable.

**Figure 1:**
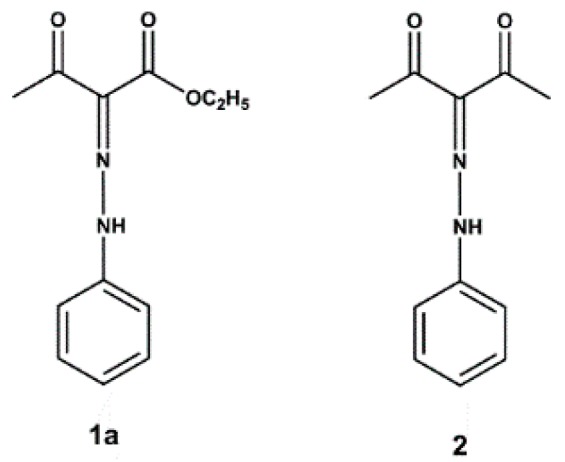
Structure of phenylhydrazinylidene derivatives **1a** and **2**.

Starting from these considerations and in continuation of our research work on antibacterial and antibiofilm agents [18,19,20,21,22], we thought it would be of interest to obtain novel SrtA inhibitors, showing more molecular diversity than the known ones, which could interfere with Gram positive virulence mechanisms as well as the biofilm formation. In order to achieve this goal, we randomly screened 200 compounds synthesized in our laboratory hoping to obtain positive hits. A high-throughput FRET-assay that monitors the SrtA-driven hydrolysis of an internally quenched fluorescent substrate (dabcyl-QALPETGEE-edans) was used to test the compounds and select sortase- specific inhibitors [16]. Among the 200 screened compounds, the most active were the phenylhydrazinylidene derivatives **1a** and **2**, where **1a**, with an IC_50_ value of 192 µM, was selected as a hit (Figure 1).

## 2. Results and Discussion

### 2.1. Chemistry

**Scheme 1:**
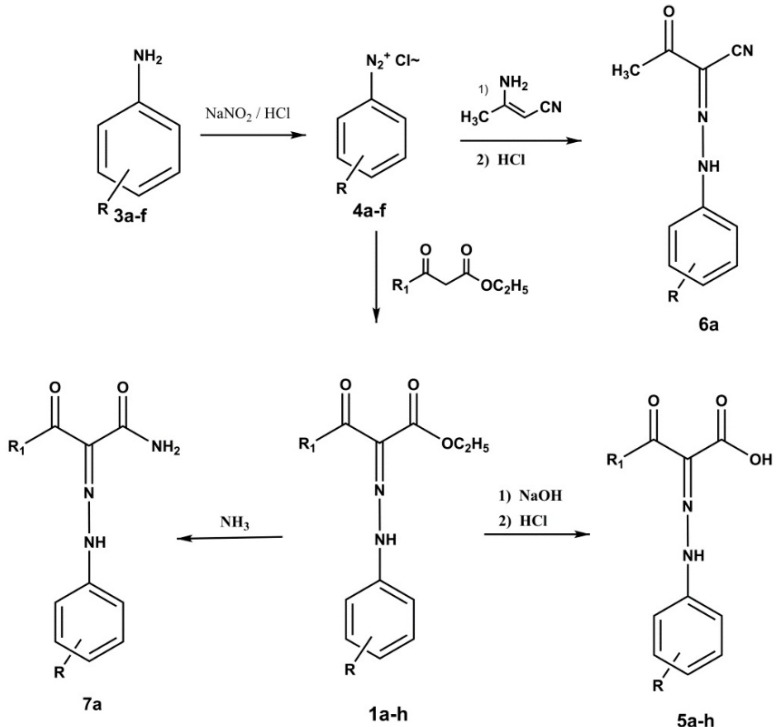
Synthetic route for compounds **1a**–**h**, **5a**–**h**, **6a** and **7a**.

A new set of compounds **1b**–**f**, analogs of compound **1a**, were prepared. The phenyl ring of **1b**–**f** bears different substituents in order to obtain a good discrimination as regards hydrophobic, electronic and steric effects. We additionally synthesized compounds **5a**–**f**, in which the carboxyethyl group of **1a**–**f** is replaced with a carboxy group and **6a** and **7a**, which bear a carboxyamido or cyano group replacing the carboxyethyl group of **1a**. Finally, analogues **1g**,**h** and **5g**,**h** were prepared, in which the acetyl group is replaced by a benzoyl group. All the compounds were obtained as shown in Scheme 1.

The diazonium salts **4a**–**f** obtained from the anilines **3a**–**f** were reacted with ethyl acetoacetate or ethyl benzoylacetate to give the ester derivatives **1a**–**h**, which, in turn, were transformed into the corresponding acids **5a**–**h** (Scheme 1). Similarly, the analogue **6a** was obtained by reacting **4a** with 3-aminocrotononitrile. Finally, the carboxyamido derivative **7a** was obtained by reacting **1a** with ammonia.

Esters **1a**–**d**,**f**,**g**, the cyano and carboxyamido derivatives **6a** and **7a** were already described. The reported synthetic method was further modified, and the abovementioned esters **1a**–**d**,**f**,**g**, and compound **7a** were obtained.

**Figure 2:**
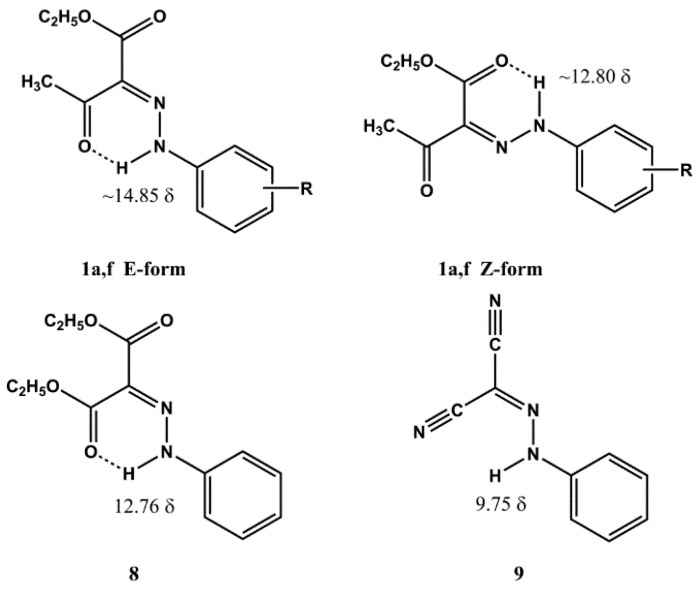
Reported NH chemical shift values of the geometrical structures of compounds **1a**,**f**, ethyl (2-phenylhydrazinylidene)mesoxalate (**8**) and 2**-**(2-phenylhydrazinylidene)propanedinitrile (**9**).

**Figure 3:**
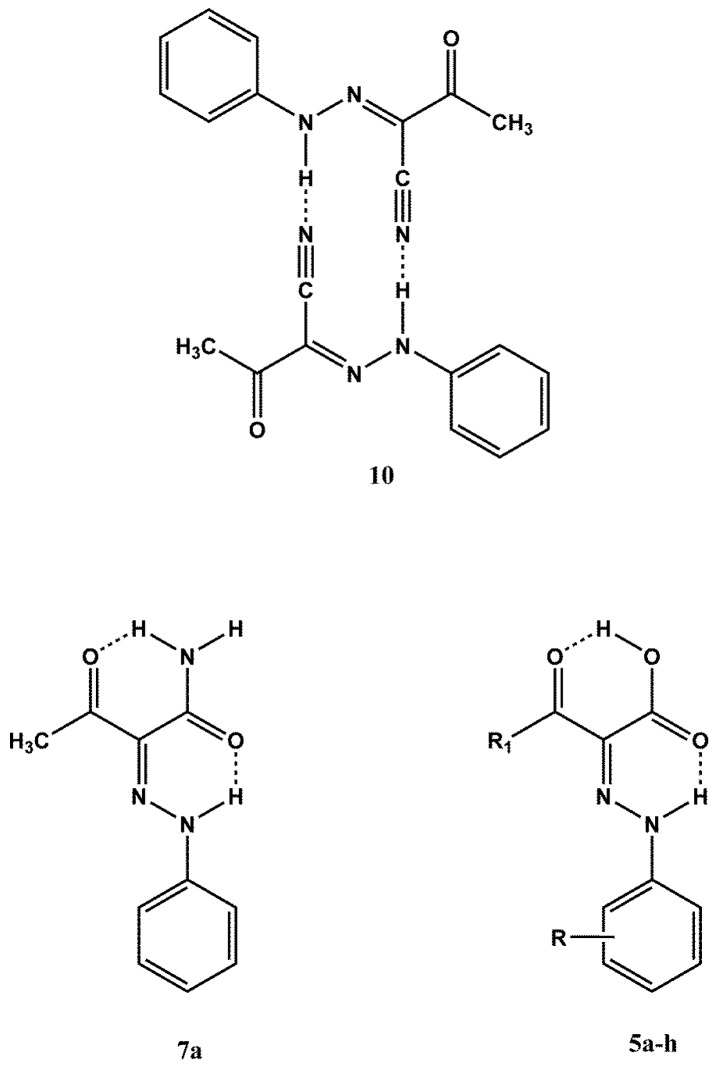
Dimeric structure **10** of (2*E*)-3-oxo-2-(2-phenylhydrazinylidene)butanenitrile (**6a**) and structures of (2*Z*)-3-oxo-2-(2-phenylazohydrazinylidene)butanamide **7a** and (2*Z*)-acids **5a**–**h**.

It is well established that the above class of derivatives exist in the arylhydrazinylidene (arylhydrazone) form [23,24], however one needs to assign the geometrical structure of the substituted C=N double bond. The structure of compounds **1a**–**d**,**f**,**g** is missing in some previously reported articles or a mixture of the *E* and *Z* forms is reported [25,26,27,28]. Moreover, opposite geometries were proposed for the same phenylhydrazinylidene derivative [29,30]. However, the crystallographically determined geometrical structure for compounds **1a**,**f** (*E* isomers) [31,32] is in agreement with that obtained by IR and ^1^H-NMR spectra [29,32]. At this point it was thought of interest to establish the geometrical structure of all the remaining compounds as this class of derivatives is not sufficiently investigated. The reported ^1^H-NMR assignment of the geometrical structures of compounds **1a**,**f** is based on the NH and CH_3_CO chemical shifts. For the compounds that bear the *E* structure, in which the NH and acetyl groups are intramolecularly bonded (see Figure 2), the NH and methyl signals are located to lower field as compared to the *Z* isomer: NH(*E*) about δ 14.85, NH(*Z*) about δ 12.80; CH_3_CO(*E*) about δ 2.6, CH_3_CO(*Z*) about δ 2.5. Considering these data, the geometrical structure of compounds **1b**–**e** was assigned on the basis of their ^1^H-NMR spectra. The esters **1b**,**c**,**e**, show in the ^1^H-NMR spectra the signal for NH in the range δ 14.61–14.90 and that for the methyl moiety of the acetyl group in the range δ 2.59–2.61, therefore the same structure of **1a**,**f** (*E* form) was assigned. As regards compound **1d**, its ^1^H-NMR spectrum shows the NH signal at δ 12.70 and the methyl one at δ 2.53, values which are compatible with the *Z* form. The geometrical structures of ethyl benzoylacetate derivatives **1g**,**h**, were assigned on the basis of the comparison between the ^1^H-NMR spectra of these compounds and that of ethyl 2-(2-phenyl-hydrazinyilidene)mesoxalate (**8**, see Figure 2) [23]. The ^1^H-NMR spectrum of compound **8** shows a singlet at δ 12.76 for the NH group intramolecularly bonded to the carboxylate one. The ^1^H-NMR spectra of **1g**,**h** show the NH signal at chemical shift values very near to δ 12.76, that is δ 12.74 and 12.60, respectively, therefore the Z structure was assigned to these compounds. The cyano derivative **6a** shows a signal in the ^1^H-NMR spectrum at δ 9.49 which excludes the H-bond of the NH with the carbonyl group. This view is supported by the ^1^H-NMR spectrum of 2-(2-phenylhydrazinylidene)propanodinitrile (**9**) [33] (see Figure 2), which shows the NH chemical shift at δ 9.57, a value very near to δ 9.49. Compound **6** might presumably be arranged to a dimeric form of type **10** where each single molecule show the *E* structure (see Figure 3). Finally, the acetoacetamide derivative **7a** exists in the *Z* form, as indicated by ^13^C- and ^15^N-NMR studies reported in the literature (see Figure 3) [34].

All the spectroscopic data of **5a**,**d**,**f** that were only partially and inaccurately reported in a previously published article [35], were *de novo* recorded and are reported herein. The ^1^H-NMR spectra of all compounds **5** showed the same pattern, *i.e.*, two distinct signals in the range δ 13.70–14.35, attributable to the NH and carboxy groups. The IR spectra showed absorptions in the range of 1697–1704 cm^−1^ for bonded carbonyl groups, and a very broad band in the 3200–2500 cm^−1^ range for the carboxy group. These values are compatible with a *Z* structure in which two strong intramolecular hydrogen bonds exist (see Figure 3). This type of structural arrangement has been confirmed by X-ray diffraction studies on compound **5a** [36].

### 2.2. Biology

The esters **1b**–**h**, the acids **5a**–**h** and compounds **6a** and **7a** were tested for their inhibitory activity, utilizing the assay monitoring enzymatic hydrolysis of sortase A FRET substrate analogue dabcyl-QALPETGEE-edans (Table 1). In accordance with preliminary data the esters **1b**–**h** were poorly active or inactive. The acids **5a**–**h** showed IC_50_ values in the range of 50–100 µM. The phenyl substitution in the phenylhydrazinylidene moiety does not play a determinant role for the activity. However, it seems that strong electron withdrawing groups in the phenyl ring, such as two chlorine atoms or a nitro group, can afford a moderate increase in the inhibitory activity in comparison to unsubstituted phenyl, possibly due to a more acidic NH group. On the contrary, the presence of electron releasing groups, such as a methyl or three methoxyl groups, produce a slight decrease of the activity. Replacement of the acetyl group with the benzoyl one in compounds **5** afforded a slight decrease of activity. Finally, the cyano derivative **6a** showed the same activity as compared to **5a**, whereas the amide **7a**, was less active than all the acids.

Tests for the antibiofilm activity of the most active SrtA inhibitors **5a**–**h**, **6a** and **7a** were performed to assess their preliminary antivirulence properties. The compounds were tested for their ability to interfere with biofilm formation of *S. aureus* ATCC 29213, *S. aureus* ATCC 25923, *S. aureus* ATCC 6538 and *S. epidermidis* RP62A at a screening concentration of 100 µM (see Table 2), a concentration at which planktonic strains were not susceptible (MIC > 270 µM). We found that all the above phenylhydrazinylidene derivatives interfered with biofilm formation. With the exception of *S. aureus* ATCC 2913, the presence of substituents in the phenyl ring of derivatives **5b**–**f**, independently of their hydrophobic and electronic properties, does not offer any advantage for the biofilm inhibitory activity. In fact, the most active compound was **5a**, showing inhibition of biofilm formation of about 60% at 100 µM against all the tested strains. A slight positive effect on the inhibition of biofilm formation of *S. aureus* 29213 strains was observed for compounds **5b**–**d**, which bear an electron-withdrawing substituent in the phenyl ring.

Replacement of the acetyl group with the benzoyl one (**5g**) in compound **5a** led to a decrease of activity for all tested strains. For the analogue compound **5h**, bearing two chlorine atoms linked to the phenyl ring**,** the antibiofilm activity was slightly increased only against the *S. aureus* 6538 strain. Finally, replacement of the carboxy group of **5a** with the cyano and carboxamide groups that resulted respectively in derivatives **6a** and **7a**, allowed only to obtain the analogues less active than **5a**.

## 3. Experimental Section

### 3.1. Chemistry

#### 3.1.1. General

All commercial chemicals were purchased from Aldrich (Sigma-Aldrich, St. Louis, MO, USA). Reaction progress was monitored by TLC on silica gel plates (Merck 60, F_254_, 0.2 mm, Merck spa, Vimodrone, Italy) and visualization on TLC was achieved by UV light. Organic solutions were dried over Na_2_SO_4_. Evaporation refers to the removal of solvent on a rotary evaporator under reduced pressure. All melting points were determined on a Büchi 530 capillary melting point apparatus (Buchi Italia srl, Cornaredo, Italy) and are uncorrected. IR spectra were recorded with a Spectrum RXI FT-IR System spectrophotometer (Perkin Elmer Italia spa, Milano, Italy) as solids in KBr discs. UV spectra were recorded with a Cary 50 Scan UV-Visible Spectrophotometer (Varian Medical System Italia, Cernusco sul Naviglio, Italy). ^1^H-NMR and ^13^C-NMR spectra were recorded in CDCl_3_ at 300.13 and 75.47 MHz respectively, using an AC series 300 MHz spectrometer (Bruker, Milano, Italia; tetramethylsilane as the internal standard): chemical shifts are expressed in δ values (ppm). Microanalyses data (C, H, N) were obtained by an Elemental Vario EL III apparatus (Elemetal Analysensysteme, Hanau, Germany) and are within ±0.4% of the theoretical values. Yields refer to products after crystallization. The name of the compounds was obtained using the ACD/Chem Sketch FREEWARE ver. 14.00 (ACD/Lab, Toronto, ON, Canada).

Physicochemical and spectroscopic data are also reported for the previously reported derivatives **1b**–**d**,**f**,**g**. Spectroscopic data allow for the correct assignment of the geometry. The synthetic procedure and a more detailed physicochemical and structural characterization of compound **7a** are also reported as only ^13^C- and ^15^N-NMR data of this compound were previously reported [34].

Some ^1^H-NMR and ^13^C-NMR spectras were presented in Supplementary Materials.

#### 3.1.2. General Procedure for the Synthesis of Compounds **1a**–**h**

The appropriate aniline (0.012 mol) in 5 N aqueous HCl solution (6 mL) was diazotized under stirring at 0–5 °C by addition of a solution of NaNO_2_ (0.88 g in 3 mL of water). After 10 min a saturated NaOAc aqueous solution was added until pH 5, followed dropwise by that of ethyl acetoacetate (1.53 mL) and NaOAc (1.44 g in 2.4 mL water) in ethanol (9 mL), maintaining the temperature under 10 °C. The reaction mixture was stirred at 5–10 °C for 30 min then at room temperature for 90 min. The precipitate of the arylhydrazinylidene derivative was filtered off, washed with water and crystallized from the appropriate solvent or purified by preparative TLC (silica gel plate, layer thickness 2 mm).

*Ethyl (2E)-3-Oxo-2-(2-phenylhydrazinylidene)butanoate* (**1a**). Yield 91%; m.p.: 78–80 °C (MeOH), lit. [29] 80 °C (MeOH), spectroscopic data are in accord with those reported in the literature [25,29].

*Ethyl (2E)-3-Oxo-2-(2-(3,4-dichlorophenyl)hydrazinylidene)butanoate* (**1b**). Yield 62%; m.p.: 100–102 °C (cycloexane), lit. [27] 71 °C (ethanol, *E*/*Z* mixture); IR ν[cm^−1^]: 3157 (NH), 1708 (CO); ^1^H-NMR (CDCl_3_) δ [ppm]: 1.40 (t, 3H, CH_3_, *J* = 7.2 Hz), 2.59 (s, 3H, CH_3_), 4.35 (q, 2H, CH_2_, *J* = 7.2 Hz); 7.19–7.55 (m, 3H, aromatic protons); 14.60 (s, 1H, exchangeable with D_2_O, NH); ^13^C-NMR (CDCl_3_) δ [ppm]: 14.31 (CH_3_); 30.82 (CH_3_); 61.26 (CH_2_); 115.52 (CH); 117.84 (CH); 127.06 (C); 128.76(C); 131.14 (CH); 133.77 (C); 141.21 (C); 164.53 (COOC_2_H_5_); 197.43 (CH_3_CO).

*Ethyl (2E)-3-Oxo-2(2-(3-chlorophenyl)hydrazinylidene)butanoate* (**1c**). Yield: 45%; m.p.: 70–72 °C (cyclohexane), lit. [26] 83–85 °C, (crude ***E***/*Z* mixture); IR ν[cm^−1^]: 3100 (NH), 1703 (CO); UV (ethanol) λ_max_ [nm], Log ε: 234, 4.14; 350, 4.25; ^1^H-NMR (CDCl_3_) δ [ppm]: 1.42 (t, 3H, CH_3_, *J* = 7.2 Hz), 2.59 (s, 3H, CH_3_), 4.34 (q, 2H, CH_2_, *J* = 7.2 Hz), 7.11–7.47 (m, 4H, aromatic protons), 14.63 (s, 1H, exchangeable with D_2_O, NH). ^13^C-NMR (CDCl_3_) δ [ppm]: 14.31 (CH_3_); 30.81 (CH_3_); 61.13 (CH_2_); 114.53 (CH); 116.28 (CH); 125.45 (CH); 126.64 (C); 130.49 (CH); 135.50 (C); 142.85 (C); 164.67 (COOC_2_H_5_); 197.26 (CH_3_CO).

*Ethyl (2Z)-3-Oxo-2(2-(4-nitrophenyl)hydrazinylidene)butanoate* (**1d**). Yield: 70%; m.p.: 120–122 °C (ethanol), lit. [26] 127.5–128 °C, (crude *E*/*Z* mixture); IR ν[cm^−1^]: 3156 (NH); 1684 (CO). ^1^H-NMR (CDCl_3_) δ [ppm]: 1.42 (t, 3H, CH_3_, *J* = 7.2 Hz), 2.53 (s, 3H, CH_3_), 4.40 (q, 2H, CH_2_, *J* = 7.2 Hz), 7.41 (d, 2H, aromatic protons, *J* = 8.7 Hz), 8.28 (d, 2H, aromatic protons, *J* = 8.7 Hz), 12.70 (s, 1H, exchangeable with D_2_O, NH); ^13^C-NMR (CDCl_3_) δ [ppm]: 14.03 (CH_3_); 26.91 (CH_3_); 62.09 (CH_2_); 115.10 (2 × CH); 126.04 (2 CH); 130.35 (C); 143.88(C); 146.50 (C); 163.10 (COOC_2_H_5_); 194.15 (CH_3_CO).

*Ethyl (2E)-3-Oxo-2-(2-(3,4,5-trimethoxyphenyl)hydrazinylidene)butanoate* (**1e**). Yield: 20%; m.p. 78–80 °C (cyclohexane); IR ν[cm^−1^]: 3142 (NH), 1701 (CO); ^1^H-NMR (CDCl_3_) δ [ppm]: 1.40 (t, 3H, CH_3_, *J* = 7.2 Hz), 2.61 (s, 3H, CH_3_), 3.86 (s, 3H, CH_3_), 3.91 (s, 6H, 2 CH_3_), 4.34 (q, 2H, CH_2_, *J* = 7.2 Hz), 6.92 e 6.70 (s, 2H, aromatic protons), 14.84 (s, 1H, exchangeable with D_2_O, NH). ^13^C-NMR (CDCl_3_) δ [ppm]: 14.26 (CH_3_); 30.77 (CH_3_); 56.10 (2 OCH_3_); 60.87 (CH_2_); 61.07 (OCH_3_); 93.75 (2 CH); 125.53 (C); 135.89 (C); 137.73 (C); 154.08 (2 C); 164.92 (COOC_2_H_5_); 196.94 (CH_3_CO). Anal. Calc. for C_15_H_20_N_2_O_6_: C, 55.55%; H, 6.22%; N, 8.64%. Found: C, 55.25%; H, 5.92%; N, 8.39%.

*Ethyl (2E)-3-Oxo-2-(2-(4-methylphenyl)hydrazinylidene)butanoate* (**1f**). Yield: 63%; m.p.: 70–72 °C (cyclohexane), lit. [32] 80–81 °C, (*E*/*Z* mixture); IR ν[cm^−1^]: 3447 (NH); 1700 (CO); ^1^H-NMR (CDCl_3_) δ [ppm]: 1.40 (t, 3H, CH_3_, *J* = 7.2 Hz), 2.34 (s, 3H, CH_3_), 2.58 (s, 3H, CH_3_), 4.33 (q, 2H, CH_2_, *J* = 7.2 Hz), 7.18 (d, 2H, aromatic protons, *J* = 8.4 Hz), 7.32 (d, 2H, aromatic protons, *J* = 8.7 Hz), 14.90 (s, 1H, exchangeable with D_2_O, NH). ^13^C-NMR (CDCl_3_) δ [ppm]: 14.35 (CH_3_); 21.03 (CH_3_); 30.77 (CH_3_); 60.87 (CH_2_); 116.40 (2 CH); 125.48 (C); 130.09 (2 CH); 135.77 (C); 139.56 (C); 165.14 (COOC_2_H_5_); 197.07 (CH_3_CO).

*Ethyl (2Z)-3-Oxo-3-phenyl-2-(2-phenylhydrazinylidene)propanoate* (**1g**). Yield 60%; m.p. 63–65 °C (MeOH), lit. [23] 65 °C (MeOH); IR ν[cm^−1^]: 3229, 3196 (NH), 1675 (CO); ^1^H-NMR (CDCl_3_) δ [ppm]: 1.37 (t, 3H, CH_3_, *J* = 7.2 Hz), 4.37 (q, 2H, CH_2_, *J* = 7.2 Hz), 7.10–7.95 (a set of signal, 10H, 2 C_6_H_5_), 12.74 (s, 1H, exchangeable with D_2_O, NH ).

*Ethyl (2Z)-3-oxo-3-phenyl-2-(2-(3,4-dichlorophenyl)hydrazinylidene)-propanoate* (**1h**). Yield: 62%; m.p. 96–98 °C (cycloexane); IR ν[cm^−1^]: 3168, 3200 (NH), 1673 (CO); ^1^H-NMR (CDCl_3_) δ [ppm]: 1.34 (t, 3H, CH_3_, *J* = 7.2 Hz), 4.36 (q, 2H, CH_2_, *J* = 7.2 Hz), 6.98–7.94 (a set of signal, 8H, C_6_H_5_ and C_6_H_3_), 12.60 (s, 1H, NH). Anal. Calc. for C_17_H_14_Cl_2_N_2_O_3_: C, 55.91%; H, 3.86%; N, 7.67%. Found: C, 56.14%; H, 3.58%; N, 7.50%.

#### 3.1.3. General Procedure for the Preparation of Compounds **5a**–**h**

To a solution of arylhydrazinylidene derivative **1a**–**h** (5 g) in ethanol (50 mL) an aqueous solution of 5 N NaOH (12 mL) was added. The mixture was stirred at room temperature for 20 h. The collected sodium salt was washed with ethanol and solubilized in cold water. The water solution was acidified with 37% aqueous hydrochloric acid and the solid was filtered off, washed with water and recrystallized from a suitable solvent.

*(2Z)-3-Oxo-2-(2-phenylhydrazinylidene)butanoic acid* (**5a**). Yield: 68%; m.p. 155–157 °C (ethanol), lit. [35] 160–161 °C (aqueous ethanol); IR ν[cm^−1^]: 3200–2500 (NH, and OH), 1698 (CO); ^1^H-NMR (CDCl_3_) δ [ppm]: 2.60 (s, 3H, CH_3_), 7.24–7.47 (m, 3H, aromatic protons), 13.93 (s, 1H, exchangeable with D_2_O, NH), 14.09 (s, 1H, exchangeable with D_2_O, COOH). Anal. Calc. for C_10_H_10_N_2_O_3_: C, 58.25%; H, 4.89%; N, 13.59%. Found: C, 58.51%; H, 4.58%; N, 13.76%.

*(2Z)-3-Oxo-2-(2-(3,4-dichlorophenyl)hydrazinylidene)butanoic acid* (**5b**). Yield: 56%; m.p.: 218–220 °C (ethanol); IR ν[cm^−1^]: 3200–2500 (NH, and OH), 1697 (CO); ^1^H-NMR (CDCl_3_) δ [ppm]: 2.61 (s, 3H, CH_3_), 7.24–7.58 (m, 3H, aromatic protons), 13.81 (s, 1H, exchangeable with D_2_O, NH), 14.01 (s, 1H, exchangeable with D_2_O, COOH). Anal. Calc. for C_10_H_8_Cl_2_N_2_O_3_: C, 43.66%; H, 2.93%; N, 10.18%. Found: C, 43.99%; H, 2.61%; N, 10.30%.

*(2Z)-3-Oxo-2-(2-(3-chlorophenyl)hydrazinylidene)butanoic acid* (**5c**). Yield: 50%; m.p.: 148–150 °C (ethanol); IR ν[cm^−1^]: 3200–2500 (NH and OH), 1698 (CO); UV (ethanol) λ_max_ [nm], Log ε: 255, 3.91; 360, 4.01; ^1^H-NMR (CDCl_3_) δ [ppm]: 2.61 (s, 3H, CH_3_), 7.21–7.49 (m, 4H, aromatic protons), 13.83 (s, 1H, exchangeable with D_2_O, NH), 14.00 (s, 1H, exchangeable with D_2_O, OH); ^13^C-NMR (CDCl_3_) δ [ppm]: 24.57 (CH_3_), 115.03 (CH), 116.47 (CH), 124.10 (C), 126.70 (CH); 130.86 (CH), 136.23 (C), 141.87(C); 165.56 (COOH); 202.23 (CH_3_CO). Anal. Calc. for C_10_H_9_ClN_2_O_3_: C, 49.91%; H, 3.77%; N, 11.64%. Found: C, 50.25%; H, 3.82%; N, 11.89%.

*(2Z)-3-Oxo-2-(2-(4-nitrophenyl)hydrazinylidene)butanoic acid* (**5d**). Yield: 41%; m.p.: 202–204 °C (1,4-dioxane), lit. [35] 194–195 °C (aqueous ethanol); IR ν[cm^−1^]: 3200–2400 (NH and OH), 1700 (CO); ^1^H-NMR (CDCl_3_) δ [ppm]: 2.65 (s, 3H, CH_3_); 7.56 (d, 2H, aromatic protons, *J* = 9.0 Hz), 8.34 (d, 2H, aromatic protons, *J* = 9.0 Hz), 13.70 (s, 1H, exchangeable with D_2_O, NH), 14.11 (s, 1H, exchangeable with D_2_O, OH); ^13^C-NMR (CDCl_3_) δ [ppm]: 24.74 (CH_3_), 116.45 (2×CH), 125.65 (C), 125.87 (2 × CH), 145.33 (C), 145.47 (C), 164.85 (COOH), 202.27 (CH_3_CO). Anal. Calc. for C_10_H_9_N_3_O_5_: C, 47.81%; H, 3.61%; N, 16.73%. Found: C, 48.15%; H, 3.32%; N, 16.64%.

*(2Z)-3-Oxo-2-(2-(3,4,5-trimethoxyphenyl)hydrazinylidene)butanoic acid* (**5e**). Yield: 32%; m.p.: 150–152 °C (ethanol); IR ν[cm^−1^]: 3200–2500 (NH and OH); 1693 (CO). ^1^H-NMR (CDCl_3_) δ [ppm]: 2.60 (s, 3H, CH_3_); 3.87 (s, 3H, CH_3_); 3.92 (s, 6H, 2 × CH_3_); 6.70 (s, 2H, aromatic protons); 14.00 (s, 1H, exchangeable with D_2_O, NH); 14.10 (s, 1H, exchangeable with D_2_O, OH); ^13^C-NMR (CDCl_3_) (δ): 23.82 (CH_3_); 55.76 (2 × CH_3_); 60.63 (CH_3_); 93.63 (2 CH); 122.73 (C); 136.24 (C); 136.58 (C); 153.78 (2 C); 165.31 (COOH); 201.10 (CH_3_CO). Anal. Calc. for C_13_H_16_N_2_O_6_: C, 52.70%; H, 5.44%; N, 9.46%. Found: C, 52.55%; H, 5.30%; N, 9.58%.

*(2Z)-3-Oxo-2-(2-(4-methylphenyl)hydrazinylidene)butanoic acid* (**5f**). Yield: 75%; m.p.: 182–184 °C (ethanol/NNDMF), lit. [35] 198–199 °C (aqueous ethanol); IR: ν[cm^−1^]: 3200–2655 (NH and OH); 1695 (CO); ^1^H-NMR (CDCl_3_) δ [ppm]: 2.38 (s, 3H, CH_3_); 2.58 (s, 3H, CH_3_); 7.22–7.36 (dd, 4H, aromatic protons); 13.98 (s, 1H, exchangeable with D_2_O, NH); 14.08 (s, 1H, exchangeable with D_2_O, OH). ^13^C-NMR (CDCl_3_) δ [ppm]: 21.12 (CH_3_); 21.03 (CH_3_); 24.37 (CH_3_); 116.64 (2 × CH); 123.13 (C); 130.08 (2 × CH); 137.18 (C); 138.51 (C); 165.81 (COOH); 201.80 (CH_3_CO). Anal. Calc. for C_11_H_12_N_2_O_3_: C, 59.99%; H, 5.49%; N, 12.72%. Found: C, 59.97%; H, 5.35%; N, 12.66%.

*(2Z)-3-Oxo-3-phenyl-2-(2-phenylhydrazinylidene)propanoic acid* (**5g**). Yield: 35%; m.p. 136–138°C (ethanol); IR: ν[cm^−1^]: 3200–2500, multiple bands (NH and COOH); ^1^H-NMR (CDCl_3_) δ [ppm]: 7.24–7.90 (a set of signal, 10 H, 2 × C_6_H_5_), 14,28 (s, 1H, NH or COOH), 14.35 (s, 1H, COOH or NH). ^13^C-NMR (CDCl_3_) (δ): 116.88 (CH), 122.89 (C), 126.87 (CH), 127.94 (CH), 129.85 (CH), 130.70 (CH), 132.64 (CH), 136.50 (C), 140.90 (C), 166.41 (COOH), 195.65 (C_6_H_5_CO). Anal. Calc. for C_15_H_12_N_2_O_3_: C, 67.16%; H, 4.51%; N, 10.44%. Found: C, 67.15%; H, 4.88%; N, 10.37%.

*(2Z)-3-Oxo-2-(2-(3,4-dichlorophenyl)hydrazinylidene)-3-phenylpropanoic acid* (**5h**). Yield: 60% m.p. 202–204 °C (ethyl acetate); IR: ν[cm^−1^]: 3200–2400, multiple bands, NH and COOH; ^1^H-NMR (CDCl_3_) δ [ppm]: 7.11–7.88 (a set of signal, 8H, C_6_H_5_ and C_6_H_3_), 14.15 (s, 2H, NH and COOH). ^13^C-NMR (DMSO) (δ): 115.85 (CH), 117.28 (CH), 127.94 (CH), 125.42 (C), 128.66 (CH), 130.42 (CH), 130.86 (C), 131.67 (CH), 132.24 (C), 133.24 (CH), 137.30 (C), 143.06 (C), 164.13 (COOH), 190.79 (C_6_H_5_CO). Anal. Calc. for C_15_H_10_Cl_2_N_2_O_3_: C, 53.44%; H, 2.99%; N, 8.31%. Found: C, 53.45%; H, 3.19%; N, 8.36%.

#### 3.1.4. *(2E)-3-Oxo-2-(2-phenylhydrazinylidene)butanenitrile* (**6a**)

See reference [37].

#### 3.1.5. *(2Z)-3-Oxo-2-(2-phenylhydrazinylidene)butanamide* (**7a**)

A solution of ethyl (2*E*)**-**3-oxo-2-(2-(phenylhydrazinylidene)butanoate (290 mg, 1.5 mmol) in ethanol (3 mL) was treated with 30% (*w*/*v*) aqueous ammonia (9 mL) under reflux for 8 h. The mixture was allowed to stand at r.t. for 12 h. The resulting solid product was filtered off and recrystallized from cyclohexane. Yield: 30%; m.p.: 134–136 °C; IR: ν[cm^−1^]: 3181, 3220s, 3355; ^1^H-NMR (CDCl_3_) δ [ppm]: 2.54 (s, 3H, CH_3_), 5.73 (s, 1H, NH), 7.18–7.39 (a set of signal, 5H, C_6_H_5_), 9.09 (s, 1H, NH), 14.60 (s, 1H, NH). ^13^C-NMR resonance values are according with literature data [34].

### 3.2. Biology

#### 3.2.1. Enzyme Activity Assay

##### 3.2.1.1 Expression of Recombinant Sortase A

Recombinant and catalytically active sortase A with the *N*-terminal deletion of residues 1–59 and possessing C-terminal hexahistidine sequence (SrtA_ΔN59_-6His) was used for the enzyme activity assay. SrtA_ΔN59_-6His was prepared according to a slightly modified previously published method [16]. Briefly, after the IPTG induction of the *E. coli* BL21 (DE3) transformed strain, the cell pellet was collected by centrifugation and resuspended in lysis buffer, and the recombinant protein was purified by affinity chromatography on a Ni-NTA column (Qiagen spa, Milano, Italy). The enzyme was eluted with an imidazole gradient and the fractions containing the protein were further purified by the gel filtration. A Superdex 75 (GE Healthcare srl, Milano, Italy) column, which was equilibrated with 10 mM sodium phosphate (pH 7.0) buffer containing 100 mM NaCl and 1 mM DTT, was used for the final purification. The fractions containing the protein were collected and concentrated to 10 mg/mL. The purified protein was analyzed using matrix-assisted laser desorption-ionization time-of-flight (MALDI-TOF) mass spectroscopy and SDS-PAGE Coomassie Blue staining.

##### 3.2.1.2. HTS Screening of Candidate Compounds and Secondary Assays of the Selected Hits and Synthesized Analogues **1a**–**h**, **2a**–**h**, **6a** and **7a**

All of the compounds were prepared as 10 mM stock solutions in dimethyl sulfoxide (DMSO) and used for the IC_50_ determination. The compounds were screened at a single dose of 100 µM (1% DMSO) in black 384-well plates (Greiner Bio-One, Kremsmunster, Australia). Two known sortase inhibitors, phenyl vinyl sulfone [38] and 1-(3,4-dichlorophenyl)-3-(dimethylamino)- propan-1-one [15], were used as the positive controls. The inhibitory activity of all of the compounds was assessed by quantifying the increase in fluorescence intensity upon cleavage of the FRET-peptide dabcyl-QALPETGEE-edans, which was used as the sortase substrate. A previously published method [39] was used with slight modifications. Briefly, the reactions were performed in a volume of 100 µL containing 50 mM Tris-HCl, 5 mM CaCl_2_, 150 mM NaCl, pH 7.5, 10 µM *S. aureus* SrtA, 20 µM fluorescent peptide substrate dabcyl-QALPETGEE-edans, and the prescribed concentrations of the test compounds or positive controls.

The peptide substrate without the recombinant SrtA was incubated in the same manner and used as a negative control. The reactions were conducted for 24 h at 37 °C, and the fluorescence emitted with an excitation wavelength of 350 nm and an emission wavelength of 495 nm after substrate cleavage was recorded.

End-point determination of product formation was used as a criterion for the primary screening. This determination was made by measuring the total product fluorescence 24 h after the initiation of the reaction. The relative inhibition activity was determined as %I = 100% − (F_sample_/F_control_*100%), where F_sample_ is the fluorescence intensity of the well containing the corresponding test compound and F_control_ is the fluorescence of the positive control reaction without inhibition.

For the IC_50_ determination, 10 μM *S. aureus* sortase A was preincubated in the reaction buffer with increasing concentrations of the inhibitory compounds (*x*–*y* μM) for 1 h at 37 °C prior to the addition of the dabcyl-QALPETGEE-edans substrate to a final concentration of 50 µM. The total fluorescence was recorded at 1-min intervals for 1 h, and the progress curves were constructed. The initial velocities of the biphasic reactions were obtained through nonlinear regression, as previously described [40,41]. The IC_50_ values were determined by fitting the obtained data to the following default four-parameter variable slope sigmoidal function in SigmaPlot 12.5 using a nonlinear least squares algorithm:

(1) y=min+(max−min)1+(xIC50)−HillSlope

### 3.3. Antibacterial and Antibiofilm Evaluation

#### 3.3.1. Microbial Strains

The reference strains *S. aureus* ATCC 29213, *S. aureus* ATCC 25923, *S. aureus* ATCC 6538 and *S. epidermidis* RP62A were used in the assays.

#### 3.3.2. Minimum Inhibitory Concentrations (MIC)

MICs of phenylhydrazinylidene-derivatives were determined by a micro-method as previously described [42]. Tryptic Soy Broth (TSB, Sigma-Aldrich srl, Milano, Italy) containing 2% glucose was used as medium.

#### 3.3.3. Biofilm Capability Evaluation (Safranin Method)

The staphylococcal strains were tested for their ability to form biofilms. Briefly, bacteria were grown in TSB containing 2% glucose overnight at 37 °C in a shaking bath and then diluted 1:200 to a suspension with optical density (OD) of about 0.040 at 570 nm [21]. Polystyrene 24-well tissue culture plates were filled with 1 mL of diluted suspension and incubated for 24 h at 37 °C. The wells were then washed three times with 1 mL of sterile phosphate-buffered saline (PBS) and stained with 1 mL of safranin 0.1% *v*/*v* for 1 min. The excess stain was removed by placing the plates under running tap water. Plates were dried overnight in an inverted position at 37 °C. Safranin-stained adherent bacteria in each well were re-dissolved to homogeneity in 1 mL of 30% *v*/*v* glacial acetic acid, and the OD was read at 492 nm. Each assay was performed in triplicate and repeated at least twice.

#### 3.3.4. Interference with Biofilm Formation Assay

The procedure described above was used to evaluate the activity of phenyl-hydrazinylidene-derivatives in the prevention of biofilm formation. Polystyrene 24-well tissue culture plates were filled with 1 mL of diluted bacterial suspension, obtained and diluted as previously described, and a concentration of 100 µM of each compound was directly added to the bacterial suspension at time zero and incubated at 37 °C for 24 h. The wells were then washed and stained with safranin as per the biofilm forming assay. By comparing the average optical density (O.D.) of the growth control wells with the sample wells, the following formula was used to calculate the percentages of inhibition for each concentration of the sample:

(2) $$\text{Inhibition }\left(\%\right)=\frac{\text{OD growth control}-\text{OD sample}}{\text{OD growth control}}\times 100$$

Each assay was performed in triplicate and assays were repeated at least twice.

## 4. Conclusions

In conclusion, we have synthesized a novel class of small molecules displaying micromolar inhibitory activity against *S. aureus* SrtA. These derivatives were screened for their activity against the enzyme using a FRET assay. Structure-activity relationship studies on compound **1a** (IC_50_ = 192 µM) have led us to obtain a more active derivative **5b** (IC_50_ = 50 µM) that could be the subject of further studies for the development of new anti-virulence agents.

## Figures and Tables

**Table:** 

3,4	a	b	c	d	e	f
**R**	H	3-4,Cl_2_	3-Cl	4-NO_2_	3,4,5-OCH_3_	4-CH_3_

**Table:** 

1,5,6,7	a	b	c	d	e	f	g	h
**R**	H	3-4,Cl_2_	3-Cl	4-NO_2_	3,4,5-OCH_3_	4-CH_3_	H	3-4,Cl_2_
**R_1_**	CH_3_	CH_3_	CH_3_	CH_3_	CH_3_	CH_3_	C_6_H_5_	C_6_H_5_

**Table 1:** Activity as sortase A inhibitors of compounds **1a**–**h**, **5a**–**h**, **6a**, **7a**.

Entry	Compounds	IC_50_ µM
1	**1a**	192
2	**1b**	ns
3	**1c**	ns
4	**1d**	100
5	**1e**	110
6	**1f**	ns
7	**1g**	ns
8	**1h**	ns
9	**5a**	80
10	**5b**	50
11	**5c**	87
12	**5d**	57
13	**5e**	92
14	**5f**	100
15	**5g**	98
16	**5h**	100
17	**6a**	80
18	**7a**	120
19	**PVS** ^1^	736
20	**BC** ^2^	120
21	**DPDAP** ^3^	10

^1^ Phenyl vinyl sulfone; ^2^ Berberine chloride; ^3^ 1-(3,4-dichlorophenyl)-3-dimethylamino-1-propanone; ns = not significant; IC_50_ ≥ 500 µM.

**Table 2:** Inhibition of biofilm formation of compounds **5a**–**h**, **6a** and **7a** at 100 μM concentration.

Comp.	Percentages of Inhibition of Biofilm Formation			
	*S. aureus* 25923	*S. aureus* 29213	*S. aureus* 6538	*S. epidermidis* RP62A
**5a**	68.3 ± 2.0	62.3 ± 1.5	60.1 ± 1.7	61.5 ± 1.2
**5b**	39.2 ± 1.4	71.3 ± 6.4	22.0 ± 0.9	29.1 ± 1.4
**5c**	41.1 ± 0.7	69 ± 4.6	30.3 ± 0.4	22.9 ± 1.3
**5d**	45.3 ± 1.7	73.7 ± 0.8	53.6 ± 1.8	48.6 ± 1.6
**5e**	28.8 ± 2.9	34.8 ± 1.3	48.2 ± 0.9	27.1 ± 1.2
**5f**	33.8 ± 2.7	40.8 ± 2.1	40.3 ± 1.9	24.4 ± 0.9
**5g**	36.8 ± 2.1	36.9 ± 1.2	41.9 ± 1.7	23.7 ± 0.7
**5h**	55.9 ± 2.6	42.7 ± 3.7	70.1 ± 2.1	45.4 ± 1.6
**6a**	45.1 ± 2.9	46.2 ± 2.3	51 ± 0.9	34.8 ± 1.8
**7a**	42.1 ± 1.8	48.2 ± 2.9	42.8 ± 1.1	45.9 ± 2.1

## Supplementary Materials

Supplementary materials can be accessed at: http://www.mdpi.com/1420-3049/21/2/241/s1.
