# Supplementary material for: Discovery of a New Class of Sortase A Transpeptidase Inhibitors to Tackle Gram-Positive Pathogens: 2-(2-Phenylhydrazinylidene)alkanoic Acids and Related Derivatives
Source: Molecules. 2016 Feb 19;21(2):241. doi: 10.3390/molecules21020241 (PMC6273394; doi:10.3390/molecules21020241)
Supplement: Supplementary file 1 [file molecules-21-00241-s001.pdf]

Discovery of a New Class of Sortase a Transpeptidase Inhibitors to Tackle Gram-Positive Pathogens: 2-(2-Phenylhydrazinylidene)alkanoic Acids and Related Derivatives

Benedetta Maggio, Demetrio Raffa, Maria Valeria Raimondi, Stella Cascioferro, Fabiana Plescia, Domenico Schillaci, Maria Grazia Cusimano, Ainars Leonchiks, Dmitrijs Zhulenkovs, Livia Basile, Giuseppe Daidone

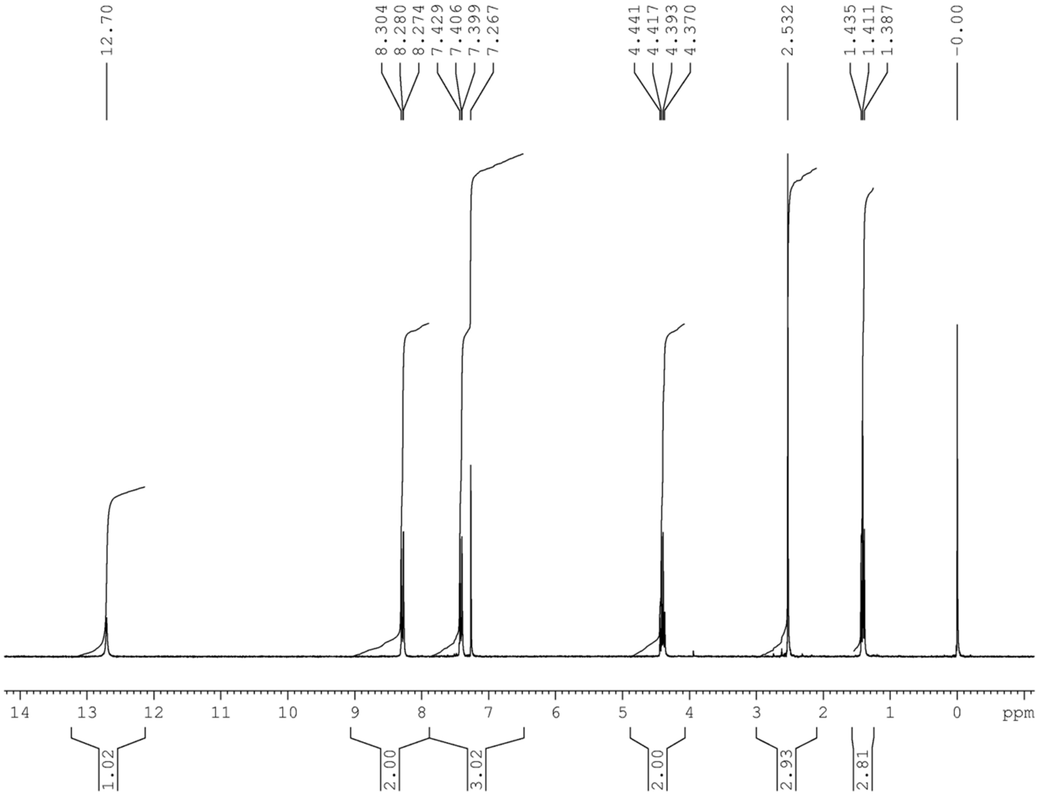

Figure S1. ^{1}H-NMR of Ethyl (2Z)-3-oxo-2(2-(4-nitrophenyl) hydrazinylidene)butanoate **1d.**

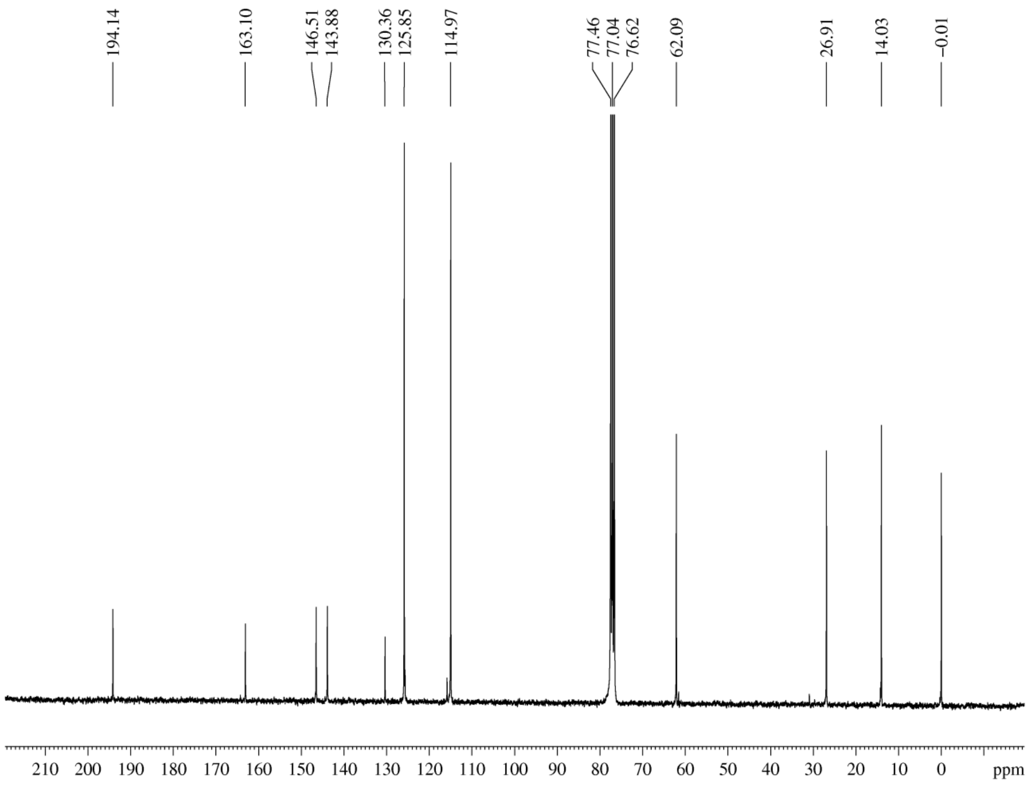

Figure S2. ^{13}C-NMR of Ethyl (2*Z*)-3-oxo-2(2-(4-nitrophenyl) hydrazinylidene)butanoate **1d**.

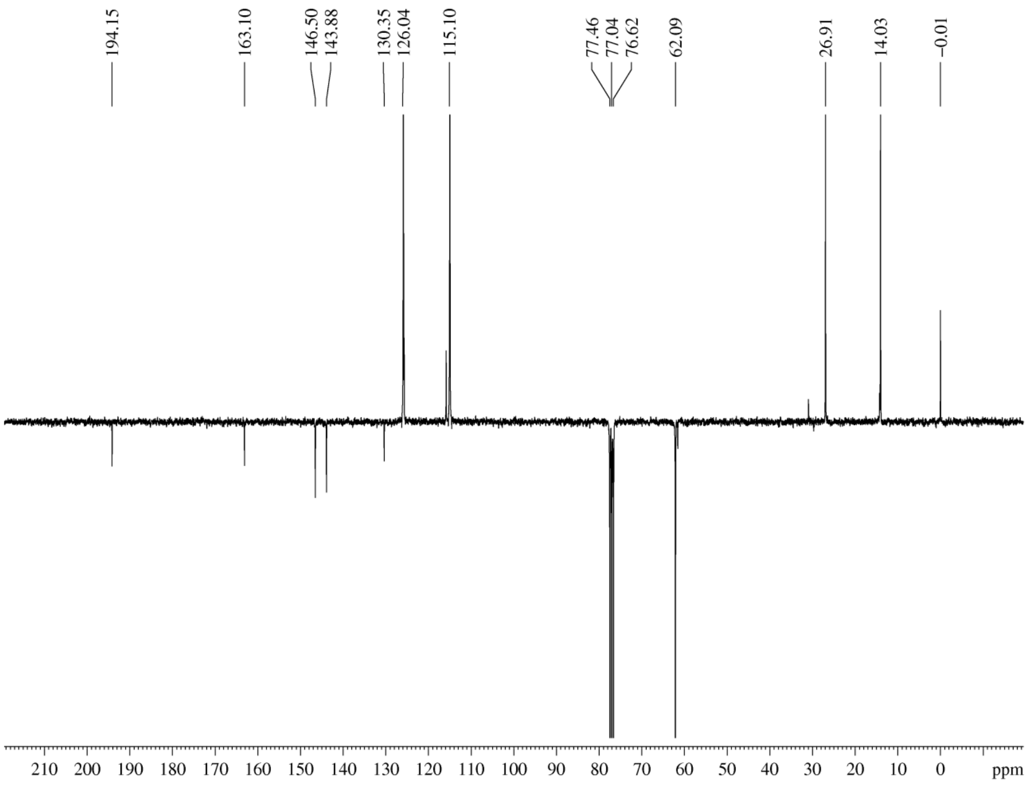

Figure S3. APT of Ethyl (2*Z*)-3-oxo-2(2-(4-nitrophenyl) hydrazinylidene)butanoate **1d**.

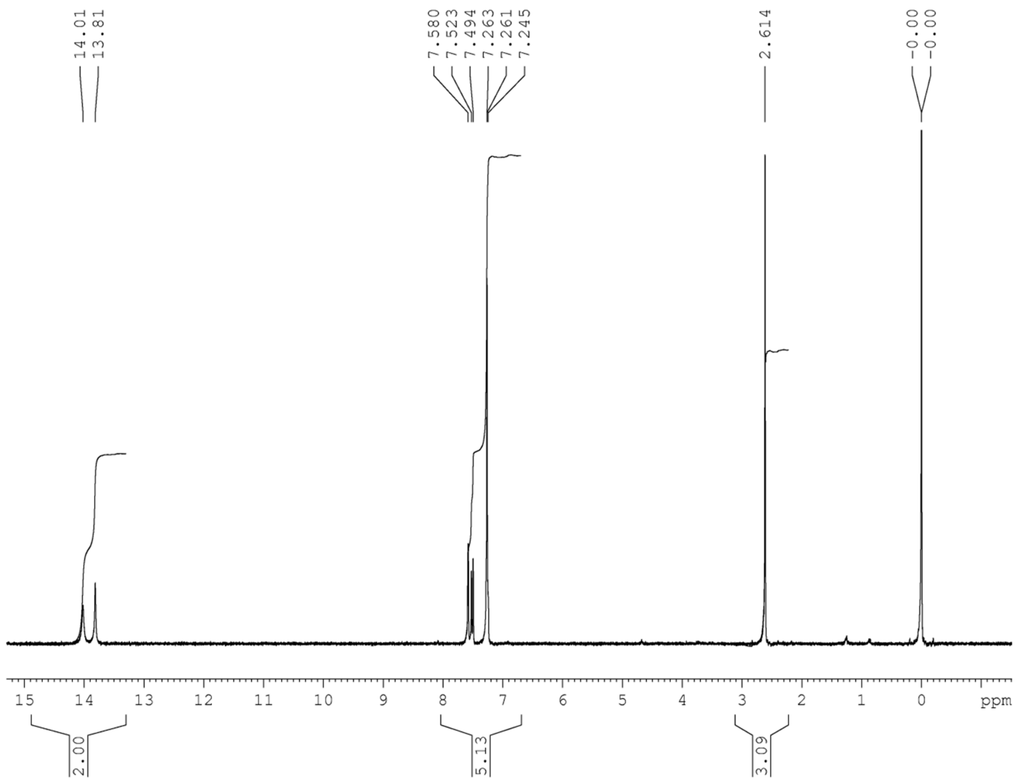

Figure S4. ^{1}H-NMR of (2-*Z*)-3-oxo-2-(2-(3,4-dichlorophenyly)hydrazinylidene)butanoic acid **5b**.

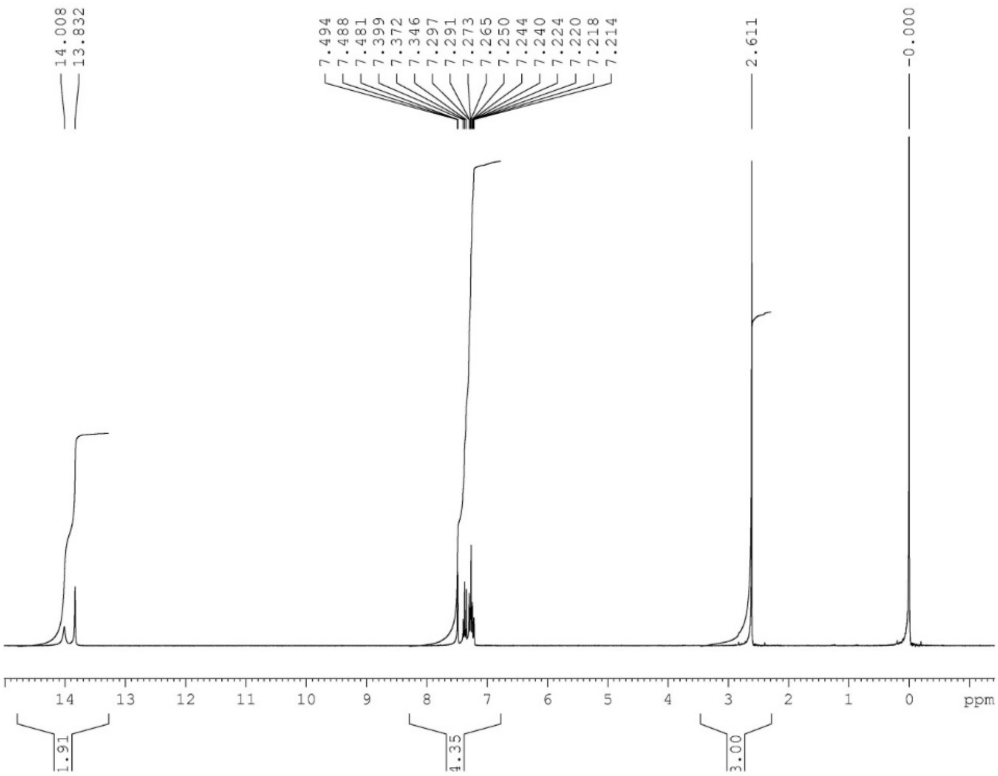

Figure S5. ^{1}H-NMR of (2Z)-3-oxo-2-(2-(3-chlorophenyly)hydrazinylidene)butanoic acid **5c**.

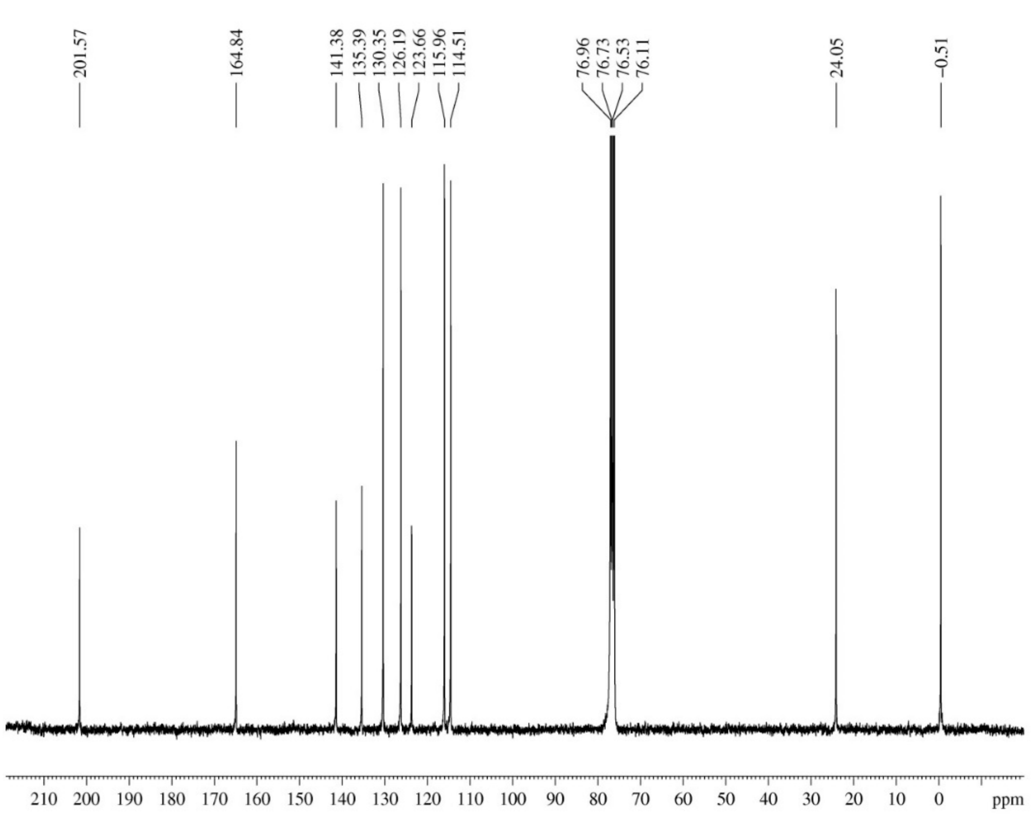

Figure S6. ^{13}C-NMR of (2Z)-3-oxo-2-(2-(3-chlorophenyly)hydrazinylidene)butanoic acid **5c**.

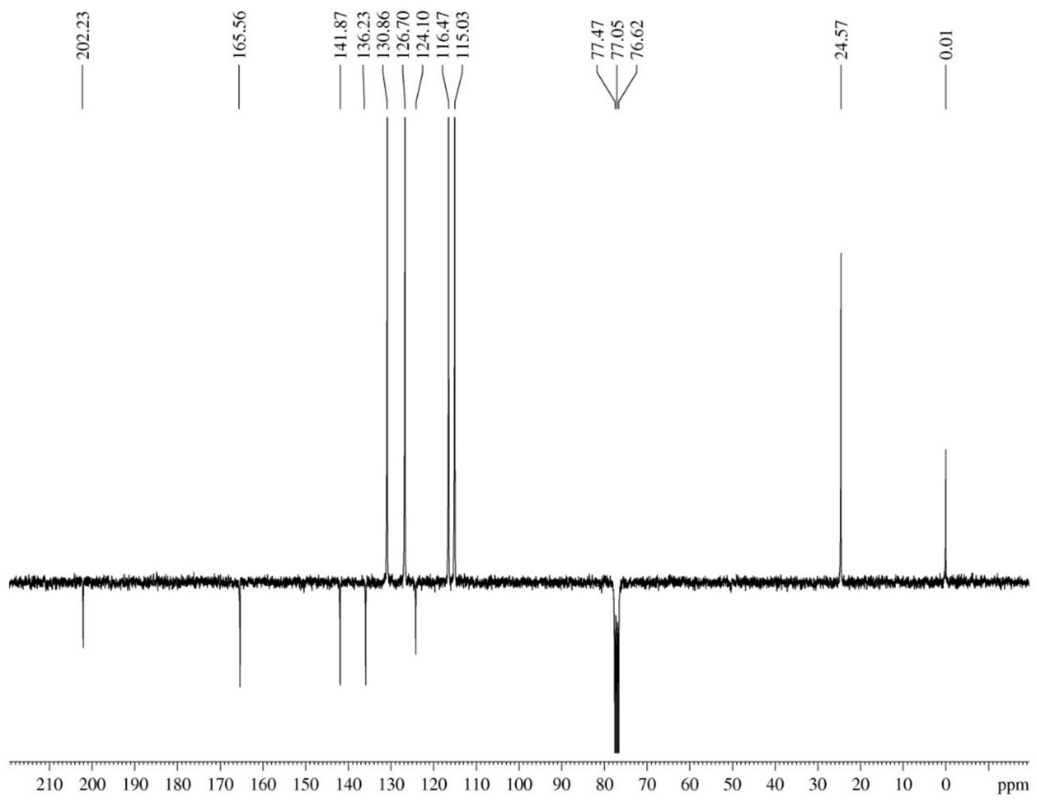

Figure S7. APT of (2Z)-3-oxo-2-(2-(3-chlorophenyly)hydrazinylidene)butanoic acid **5c**.

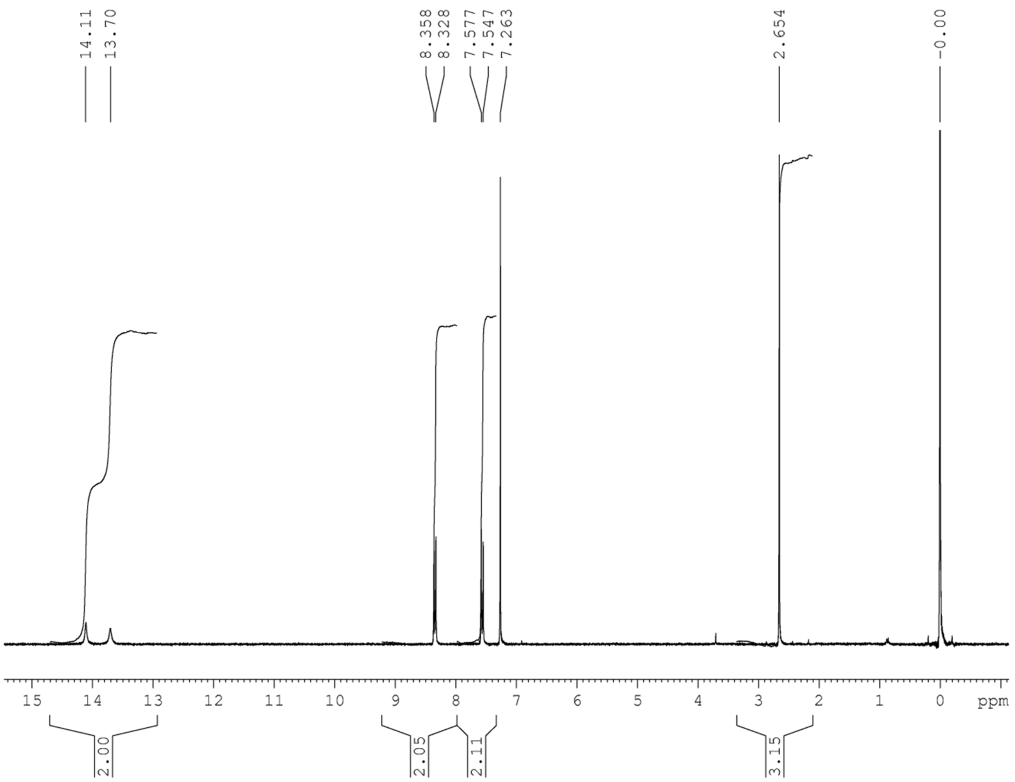

Figure S8. ^{1}H-NMR of (2Z)-3-oxo-2-(2-(4-nitrophenyly)hydrazinylidene)butanoic acid **5d**.

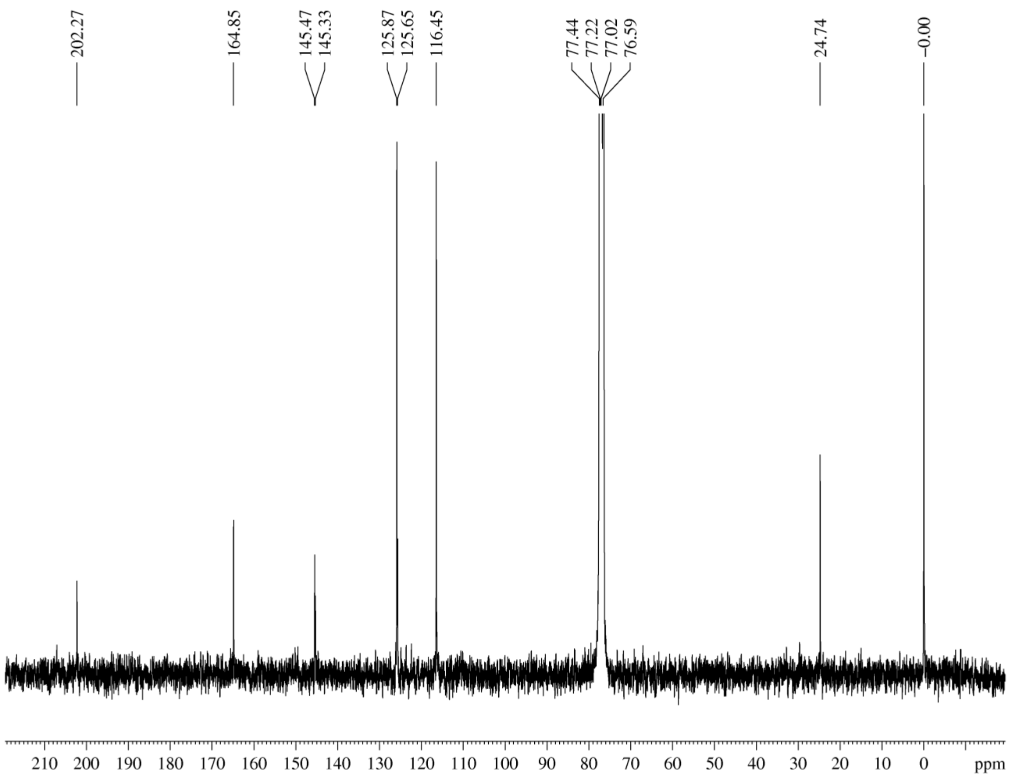

Figure S9. ^{13}C-NMR of (2*Z*)-3-oxo-2-(2-(4-nitrophenyly)hydrazinylidene)butanoic acid **5d**.
